# Single point measurement of cystatin C has similar performance as serum creatinine for assessment of kidney function in critically ill patients

**DOI:** 10.1186/cc14369

**Published:** 2015-03-16

**Authors:** J Houthoofd, M Carlier, J De Waele, R Vanholder, J Delanghe, J Decruyenaere, E Hoste

**Affiliations:** 1 OLV Van Lourdes Hospital, Waregem, Belgium; 2 Ghent University Hospital, Ghent, Belgium

## Introduction

The gold standard for routine evaluation of kidney function is measurement of serum creatinine concentration (Scr). In ICU patients, muscle loss and dilution leads to decreased Scr. Scr is distributed in total body water, resulting in delay of Scr changes when the glomerular filtration rate (GFR) changes (lag time). Cystatin C (CysC) is a protein produced by all cells with a nucleus and therefore less affected by muscle mass. Also, the lag time may be shorter as the volume of distribution is only extracellular fluid. The objective of this study was to evaluate whether single point measurement of CysC is more adequate than Scr for monitoring of kidney function in ICU patients.

## Methods

Data were collected in two prospective single-center studies on a convenience sample of ICU patients. During the 24-hour study period, we measured CysC, Scr, and urinary inulin clearance (Cinu) as a gold standard for assessment of GFR. We compared Scr and CysC, with Cinu. Also, we assessed Cinu in patients who had CysC and Scr within the normal sex and age corrected limits. Finally, we determined the ability of CysC and Scr to detect normal range and decreased GFR (80 to 120 ml/minute/1.73m^2^ resp. <60).

## Results

We included 68 patients, with median age 58 years (IQR 29 years), length of stay in the hospital before study 11 days (IQR 16), and APACHE II score 19 (IQR 9). Scr was 1.12 (IQR 1.55), CysC 0.64 (IQR 0.73), and Cinu 80 ml/minute/1.73 m^2^ (IQR 82). Cinu was markedly decreased in patients with Scr in normal range (*n *= 12) compared with patients with CysC in normal range (*n *= 22) (55 ml/minute/1.73 m^2^ (IQR 57.4) vs. 100 (IQR 42.2), *P *< 0.001). Patients with normal range Scr had similar proportion of patients with Cinu in the normal range compared with normal range CysC patients (33.3% vs. 45.5%, *P *= 0.23). ROC analysis showed that Scr and CysC had similar performance for detection of normal range Cinu (AUC: 0.66; 95% CI = 0.53 to 0.77 vs. 0.77; 95% CI = 0.65 to 0.87; *P *= 0.118), and decreased Cinu (AUC: 0.86; 95% CI = 0.76 to 0.97 vs. 0.94; 95% CI = 0.88 to 1; *P *= 0.113).

